# Impact of Deformability and Rigidity of Starch Granules on Linear and Non-Linear Rheological Behavior of Waxy Rice Starch Gels and Applicability for Food End Uses

**DOI:** 10.3390/foods13121864

**Published:** 2024-06-13

**Authors:** Ngamjit Lowithun, Leonard M. C. Sagis, Namfone Lumdubwong

**Affiliations:** 1 Department of Food Science and Technology, Faculty of Agro-Industry, Kasetsart University, Bangkok 10900, Thailand; 2 Laboratory of Physic and Physical Chemistry of Food, Department of Agrotechnology and Food Sciences, Wageningen University, Bornse Weilanden 9, 6708WG Wageningen, The Netherlands

**Keywords:** waxy rice starch, particle size, chain length, granule rigidity, non-linear viscoelasticity, Payne effect

## Abstract

The objective of this study was to investigate granule size and distribution and deformability of granules and their effect on the rheological properties of waxy starch gels. Native (granular) waxy rice gels (10%) were prepared, and their response in oscillatory shear was investigated in the linear and non-linear viscoelastic regime. The results show the gels were mainly composed of aggregated and deformed swollen granules. Significance of granule size and its distribution, deformability of granules, and the molecular characteristics of amylopectin (AP) on storage modulus of those gels was demonstrated. A low degree of deformability of granules, typical for small granules with a broad size distribution and small molecular size of AP with short external chains, resulted in rigid and brittle gels. Highly deformed granules and high AP leachates, however, yielded soft gels. It was found that the transition of elastic to plastic behavior in the non-linear regime (LAOS) was gradual when AP had long external chains, but an abrupt transition was observed with the gel with short exterior chains of AP. Differences in rheological properties of cohesive waxy starch gels appear to be mainly impacted by the varying degrees of granule deformability and rigidity, which is further attributed to a combination of factors, including granule size, particle size distribution, molecular size, the external chain length of amylopectin (AP), and lipid content. The significance of this study is that it will assist the food industry in selecting suitable waxy rice starches to gain desired textural properties of end products.

## 1. Introduction

Waxy rice (*Oryza sativa* var. *glutinosa*) is a type of rice grown predominantly in Southeast, South, and East Asia. This staple food for Asians is consumed in different forms, such as cooked grain, flour, and starch-based products (e.g., sticky rice pudding, glutinous rice balls, custards, and fillings). This particular rice starch consists almost exclusively of amylopectin (AP). It possesses high-water-holding capacity, great binding ability, a soft but tacky texture, and excellent freeze–thaw stability, all contributing to the unique paste/gel characteristics that make the waxy rice starch an excellent option for soups, sauces, baby foods, puddings, and a medium for meat marinating and injecting [1].

To determine the viscoelasticity profile of waxy rice starch (WRS) paste/gel for predicting starch behavior during food processes and determining the texture of starch-based foods, the viscosity (consistency) is commonly determined by a rapid visco analyzer (RVA). This instrument is a rotational viscometer with variable temperatures and shear forces. The typical pasting profile of WRS exhibits a high peak viscosity (PV), a large breakdown (BD), and a very low setback (SB). A 6% *w*/*w* paste of WRS displayed a cohesive texture and flowed smoothly [2,3]. Different pasting properties and texture, for example in cooked rice and sticky rice dumplings, were observed among cultivars of waxy rice starch [3,4,5,6]. Cooking quality, stickiness, adhesiveness, and overall acceptance were correlated with PV, HV, FV, BD, and SB of WRS [6]. The viscoelasticity of 25% starch gels from twenty WRS cultivars was also determined using a rheometer. The storage modulus upon cooling to 5 °C after gelatinization (G′_5 °C_) representing elasticity (stiffness) of those gels was ~33–42 Pa, and their tanδ_5 °C_ values ranged from 0.78 to 0.95. The results indicate that WRS gel networks are more liquid-like and have a weak structure, and their properties can vary significantly for different cultivars [7]. 

The effect of molecular weight and branching characteristics of AP molecules on pasting properties and viscoelasticity of waxy starches were previously demonstrated. Small molecular size of AP with higher external chain length (ECL) gave a high FV [5]. A high proportion of DP ≥ 37 was positively correlated with the values of complex moduli (G*) of 15% waxy starch gels [8]. The retrogradation of 6% waxy starch paste decreased when the starch had a high proportion of chains with DP 6–11 but a low proportion of chains with DP 12–24 [3]. 

Besides the influence of AP molecular characteristics, starch granule rigidity and the swelling of starch granules were also proposed and demonstrated as important factors contributing to the viscoelasticity of waxy starch gels [7,9,10]. A starch gel is regarded as a composite material in which swollen granules are the filler, and leachates of starch molecules, i.e., AM and AP, form the network matrix [11,12]. AP has been recognized to be responsible for granule swelling, and the morphology of the swollen granules affects the volume fraction of the dispersed phase of the composite. Waxy starch has much greater granule swelling ability than that of normal starch due to the lack of AM and lipids, which restrict granule swelling [13]. In turn, its granule rigidity is extremely low as their highly swollen granules are easy to deform and disintegrate under the influence of shear forces. In other words, waxy starch pastes/gels consist of deformed and/or disintegrated granules embedded in an amylopectin (AP) matrix [14] and the rheological behavior of the native waxy starch gel greatly depends on granule rigidity and deformability, interactions between granules, the continuous phase of solubilized AP, granule–AP interactions, and the surface of granules [11,15].

The effects of granule rigidity and swollen granules of waxy starch on the rheological properties of waxy paste/gel, depend also on starch concentration, sample preparation, cooking temperatures, and shear forces [16]. The 6% *w*/*v* gels of waxy tapioca and waxy rice were considered to be AP polymer networks since their granules did not have much impact on their rheological property [17]. Wang et al. [10] reported that an 8% *w*/*w* granular waxy maize starch gel/paste displayed a significantly higher storage modulus (G′) and a lower tan δ than their non-granular counterparts (G′ 10 Pa vs. G′ 3 Pa and tan δ 0.65 vs. tan δ 1.3, respectively). In contrast, Precha-Atsawanan et al. [9] demonstrated a significant effect of waxy granules on the rheological properties of waxy starch gels. The 15% *w*/*v* debranched (DB) waxy rice starch (WRS) gel, which consisted of clusters of stiff polymers, was much stronger and more brittle than the native WRS gel prepared from the same solid content, when determined in the linear viscoelastic regime (LVE).

It is well-known that particle shape, size, rigidity, and distribution significantly affect viscoelasticity of composites. Although the size of rice granules are the smallest among cereal species [18], variations in particle size and distribution of WRS were observed among cultivars [7,18]. Differences in granule rigidity and swollen phase volume of waxy starches were suggested as other factors contributing to differences in moduli and gel stiffness between cultivars [7]. 

Based on previous studies, the presence of disintegrated waxy granules in the composite system seemingly had a significant effect on the rheological properties of waxy gels. To the best of our knowledge, there have not been any studies investigating the impact of granule size and distribution, rigidity, and deformability on properties of waxy rice starch pastes/gels. Four certified cultivars of waxy rice provided by the Thai Rice Department were chosen for this study. RD6 is a recommended cultivar grown domestically due to its good cooking quality. However, the cultivar is susceptible to blast disease and bacterial leaf blight when grown at higher elevations. HY71 has an inferior cooking quality than that of RD6 but it is resistant to blast disease. RD12 is a cross of RD6 and HY71. The cultivar is resistant to blast disease, and has a shorter harvest period, but gives an equivalent cooking quality to RD6. SMJ is an indigenous cultivar that is resistant to blast disease and bacterial leaf blight. Its cooking quality is similar to that of RD10 [19]. The objective of this study was to investigate granule size and distribution and deformability of granules and their effect on the rheological properties of waxy starch gels. Their linear and non-linear rheological response in oscillatory shear (SAOS and LAOS) of 10% *w*/*v* WRS gels/pastes were determined. Their granule size and distribution and changes in microstructure including swollen granules and deformability were studied and linked to the rheological behavior.

## 2. Materials and Methods

### 2.1. Materials

Four milled waxy rice cultivars (RD6, RD12, HY71, and SMJ) were obtained from the Rice Seed Division, Rice Department, Ministry of Agriculture and Cooperative, Thailand. The samples were harvested in 2021 at the rice stations in Sakhon Nakorn, Chiang Mai, Nong Khai, and Chiang Rai provinces. The collected mature seeds were air-dried, dehulled, polished, and packed in sealed polyethylene bags, and stored at 4 °C until further analyses. 

The waxy rice starch (WRS) was isolated from wet-milled rice flour using the alkaline extraction method, chosen for its efficiency of protein removal and being the industrial extraction process [20]. The concentration of NaOH and the stirring used in the study were previously tested and verified that waxy rice starch granules were not damaged. Rice flour (200 g) was mixed with 0.05 M NaOH (500 mL). The slurry was stirred gently using a magnetic bar for 3 h at room temperature, and stored overnight at 8 °C. Subsequently, the slurry was filtered through a 75 μm mesh sieve and centrifuged at 3000× *g* for 20 min. The supernatant and a gray layer on top of the sediment were discarded. The residue was washed twice with distilled water and centrifuged. The starch was then mixed with distilled water. The slurry was adjusted to pH 7 with 1 M HCl, centrifuged, and washed three times with distilled water. The starch was dried in a hot-air oven at 40 °C for 24 h, gently ground with a pestle and mortar, and sieved through a 100-mesh sieve. The starch samples were packed in polyethylene bags and stored at 4 °C until further analyses.

### 2.2. Particle Size Distribution

Particle size distributions of waxy rice starches were analyzed by static light scattering. Rice starch (1% wt.) was dispersed in water with a vortex mixer and then characterized using a Mastersizer 3000 particle size analyzer with Hydro LV (Malvern Instruments Ltd., Worcestershire, UK). The starch solution was injected into circulating water with 1000 rpm paddle speed, and sonicated 45 s prior to measurement. A refractive index of 1.54 for starch and 1.33 for water, and non-spherical mode were used for analysis. The particle size was expressed as median diameter of volume distribution (D50) and surface weight mean (D[3,2]). The polydispersity index of size distribution was calculated as (D90–D10)/D50.

### 2.3. Chemical Compositions, Amylose Content, and Iodine Absorptivity Spectra

Moisture content, protein, ash, and fat contents of starches were determined according to the methods of AACC [21] and AOAC [22]. The apparent amylose (AM) content of starches was measured using the iodine-binding capacity method [23]. The maximum wavelength (λ-max) and the absorption spectra of their amylose–iodine complex were analyzed. Starch (30 mg) was dissolved in 2 M NaOH (1 mL) by mixing with a vortex mixer. Then distilled water (2 mL) was added to the solution. To the solution (100 μL), 5% trichloroacetic acid (5 mL) and 0.01 N I_2_-KI solution (0.127 g I_2_ and 0.3 g KI/100 mL) was added. The absorbance profile of samples was determined using a UV–vis spectrophotometer (Model Lambda 265, Perkin Elmer, Seoul, Republic of Korea) with UV Lab 4.1.3 software. The wavelength was scanned from 400 to 900 nm and water was used as a blank. The λ-max, absorptivity, and slope ratios of the absorbance spectrums were analyzed following Knutson [24]. The slopes of absorbance spectrums were calculated using Origin 2018 software (see calculation method in Supplementary Materials).

### 2.4. Analysis of Fine Structures

The number average degree of polymerization ($\bar{{DP}_{n}}$) was determined using the modified Park–Johnson method [25], which is commonly used for analysis of reducing groups in carbohydrates with the ferricyanide reduction method, and the phenol–sulfuric acid method [26] for total carbohydrates. The $\bar{{DP}_{n}}$ was calculated from the amount of total carbohydrates divided by the amount of reducing end in the sample. β-amylolysis of AP was analyzed according the method of Zhu and Bertoft [27] with some modifications. AP (2.5 mg/mL) was redissolved by boiling for 1 h. To the solution (0.5 mL), 0.1 M sodium acetate buffer (0.495 mL, pH 4.8) and β-amylase (5 μL) were added. The mixture was incubated in a water bath with shaker at 37 °C for 5 h. The colorimetric Somogyi–Nelson method [28] based on color complex between copper oxidized sugar and arsenomolybdate reagent was applied to determine the amount of maltose hydrolyzed from the reaction (see calculation method in Supplementary Materials). The intrinsic viscosity [η] of AP in water was determined using an ubbelohde viscometer as in the method described by Kowittaya and Lumdubwong [5] (see calculation method in Supplementary Materials).

The debranched AP was prepared following a method of Zhu et al. [29] with modifications. Waxy starches or AP (10 mg) were dissolved in 90% DMSO (400 µL) by heating at 80 °C for 1 h and then stirring overnight at room temperature. The solution was mixed with ethanol (8 mL) and then centrifuged at 4000× *g* for 10 min. The precipitated sample was redissolved in distilled water (3.6 mL) and heated at 95 °C for 1 h. Sodium acetate buffer (400 µL, 0.1 M, pH 5.5), isoamylase from *Pseudomonas* sp. (2 µL, specific activity 240 U/mg), and pullulanase from *Klebsiella planticola* (2 µL, specific activity 30 U/mg) were added after cooling. The mixture was incubated in a shaker water bath at 37 °C for 48 h, followed by boiling for 10 min to deactivate the enzymes. The debranched sample was diluted to a concentration of 1 mg/mL and filtered through a 0.45 µm PTFE membrane filter. The average chain length ($\bar{CL}$) of debranched amylopectin was calculated by dividing the value for total carbohydrate by its reducing value [30]. The total carbohydrate and the reducing value were determined by the phenol sulfuric acid method [26] and a modified Park–Johnson procedure [31], respectively. The external chain length ($\bar{ECL}$) was calculated from ($\bar{CL}$ × %β − amylolysis) + 2, the internal chain length ($\bar{ICL}$) was taken as $\bar{CL}$ − $\bar{ECL}$ − 1, and average number of chains per branched molecule ($\bar{NC})$ was $\bar{{DP}_{n}}$/$\bar{CL}$, where $\bar{{DP}_{n}}$ is the number average degree of polymerization.

### 2.5. Physico-Chemical Properties of Starches

Swelling power (SP) and water solubility index (WSI) of starches was determined following the method of Crosbie [32], with slight modifications. Starch (1.0 ± 0.001 g, db) was weighed in a centrifuge tube (50 mL), distilled water (30 mL) was added, and then the sample was mixed quickly with a vortex mixer. The suspension was heated in a water bath at 92.5 °C for 30 min. Each tube was inverted at regular intervals (initially 20 s intervals for 3 min, then 30 s intervals for 2 min, every 1 min for 5 min, and every 5 min). The sample was cooled rapidly in cooling water (10 °C) for 10 min and then centrifugation was applied at 4000× *g* for 20 min to sediment gels. The supernatant was removed, evaporated, and dried at 105 °C for 5 h. The water-soluble index (WSI) was calculated as the percentage ratio of the dry weight of soluble dry matter to that of starch. Swelling power was calculated as the weight of sedimented gel, divided by the weight of starch (i.e., the original dry weight of starch minus the soluble dry matter determined from the supernatant). The close packing concentration (C*) and volume fraction (ϕ) were calculated following the equations of Vandeputte et al. [33] and Lii et al. [34], respectively.

C* = (dry matter starch weight/weight of sedimented gel) × 100
(1)

ϕ = C × SP × (1 − WSI)
(2)

Here, C is the starch concentration.

Thermal properties of waxy starches were determined by a differential scanning calorimeter (DSC 1, Mettler Toledo, Zurich, Switzerland). The weight ratio of starch (dry basis) to water was 1:3, corrected for sample moisture. All analyses were performed in stainless steel pans. Gelatinization properties of samples, onset (T_o_), peak (T_p_), conclusion (T_c_) temperatures, and endothermic enthalpy (ΔH) were determined by heating from 30 °C to 140 °C at 10 °C/min.

### 2.6. Linear and Non-Linear Rheological Properties

#### 2.6.1. Preparation of Starch Suspensions

Starch suspensions (10%, *w*/*w*) were concomitantly stirred at 250 rpm using a magnetic bar and pre-heated in a water bath until the suspension was thick enough to prevent starch settling. The pre-heating temperature was ~4–5 °C below the onset gelatinization temperature (T_o_) of each starch. The suspension was transferred onto the plate of a rheometer (Model MCR502, Anton Paar, Graz, Austria), with sandblasted parallel probe and plate (50 mm diameter) and a gap size of 1.0 mm for all experiments. Paraffin oil (highly liquid, Merck, Darmstadt, Germany) was applied on the edge of the samples, and a solvent trap was used to minimize water evaporation during measurement.

#### 2.6.2. Small Amplitude Oscillatory Shear (SAOS)

Dynamic temperature sweep tests of the samples were performed by heating from 45 °C to 95 °C at a heating rate of 10 °C/min, followed by holding at 95 °C for 5 min, and then cooling to 25 °C at a cooling rate of 7.5 °C/min. An amplitude sweep test was first used to determine the range of the LVE regime, with shear strain range of 0.01–1000% with a constant frequency of 1 Hz at 25 °C. The heating and cooling rates were chosen to mimic the starch pasting temperature profile. A shear strain of 0.1% was used at constant frequency of 1 Hz for determination of the storage modulus (G′), loss modulus (G″), and loss tangent (tan δ) during heating and cooling. After the temperature sweep test, the gel was kept at 25 °C for 10 min to allow gel relaxation. Subsequently, a frequency sweep test from 0.1 to 10 Hz at 25 °C was used at fixed shear strain within the LVE regime (1%).

#### 2.6.3. Large Amplitude Oscillatory Shear (LAOS)

After the SAOS test, the samples were held for 10 min before applying a strain sweep in the non-linear regime. The amplitude sweep test was used to compare behavior at large deformations like those that occur during processing. A strain amplitude range of 0.01–1000% with a constant frequency of 1 Hz at 25 °C was used. The Payne effect (weak strain overshoot in G″) was calculated by Equation (3).

Payne height = (G″_max_ − G″_LVE_)/(G″_LVE_) (3)

where G″_max_ is the maximum value of G″, and G″_LVE_ is the G″ value in the linear viscoelastic regime.

The responses of the starch gels in LAOS tests were evaluated by Lissajous plots created using Matlab R2022b software with a home-built Matlab script. The dissipation ratio and strain stiffening ratio (S) were calculated using [35]

Dissipation ratio = (π γ_0_ G″_1_)/(4 σ_max_) (4)

S = (G′_L_ − G′_M_)/(G′_L_) (5)

where, G″_1_ is the first-order viscous Fourier coefficient, γ_0_ is the strain amplitude, and σ_max_ is the maximum stress. G′_L_ is the shear elastic modulus at large deformation, and G′_M_ is the shear elastic modulus at minimum strain.

### 2.7. Pasting Properties

Pasting properties of starch suspensions (2.7 g db, 25 mL) were determined by using a rapid visco analyzer (RVA 4500, Perten Instruments, Sydney, Australia) with a canister and a paddle rotated at 160 rpm. The pasting was performed by first holding at 50 °C for 2 min, before heating to 95 °C at a rate of 7.5 °C/min, followed by holding at 95 °C for 2 min, then cooling to 50 °C at 7.5 °C/min, and finally, holding at that temperature for 4 min [20]. 

### 2.8. Microstructure of WRS, WRS Pastes, and WRS Gels

Starch suspension (1%) was prepared by dispersing 10 mg of the sample in distilled water. Then 0.1% Nile blue A (1 μL) was added for staining for 10 min. The excess dye was washed by centrifugation at 3000× *g* for 5 min at 20 °C. The stained granules were mixed with 1 mL of 50% glycerol and loaded onto a slide and covered with a glass coverslip. 

The waxy gels at their point where G’ equals G′_max_ (the peak value in G’ in the temperature sweep) and G′_25_ (the value of G’ after cooling to 25 °C) were collected, and their microstructure was analyzed by a confocal microscope. The G′_max_ gel was cooled down to 25 °C before dye staining. The G′_max_ gel (200 mg) was stained with 0.1 *w*/*v*% Nile blue A (50 μL) and left at room temperature for 1 h. The stained gel was then loaded on a slide and covered with a glass coverslip. Additional steps were applied on the G′_25_ gel to observe deformed waxy granules more clearly. The 200 mg G′_25_ gel was gently mixed with distilled water (1.8 mL) in a 2 mL Eppendorf tube and was centrifuged at 16,000× *g* for 5 min at 20 °C. The sedimented gel of 100 mg was then stained with 0.1% Nile blue (25 μL) and stored for 1 h at room temperature. The excess dye was washed by centrifugation at 2000× *g* for 5 min; the washing step was repeated four times. Then 10 mg of stained gel was finally diluted with 30 μL of distilled water and was covered with a coverslip. The observation of the microstructure of those gels was determined by a He/Ne laser: CLSM (ReScan Confocal microscope, RCM1, Confocal, Amsterdam, The Netherlands) with an excitation wavelength of 640 nm with 60×/1.2 W objectives. Digital images were acquired and analyzed by a CMOS camera in combination with the Micromanager Software (version 2.0.1) and ImageJ 1.53e software (National Institute of Health, Bethesda, MD, USA).

The microstructure of waxy pastes obtained from RVA was determined by a light microscope. The samples were collected at their PV and FV points, and cooled to room temperature. Then 150 mg of each sample was stained with 100 µL of iodine solution (1 mL solution containing 1.67 mg I_2_ and 3.33 mg KI) for one and a half minutes. The stained sample was smeared on a slide and then observed under a light microscope (Axio Lab.A1, with a MicroPublisher 3.3 RTV digital camera Zeiss, Oberkochen, Germany) at a magnification of 40×.

### 2.9. Statistical Analysis

All experiments were performed in at least duplicate, and data are reported as mean values. SPSS 25 software was used for the analysis of variance (ANOVA) and Duncan multiple comparison at 5% of significance.

## 3. Results and Discussion

### 3.1. Particle Size Distribution and Morphology of Native Waxy Rice Starches

**Figure 1:**
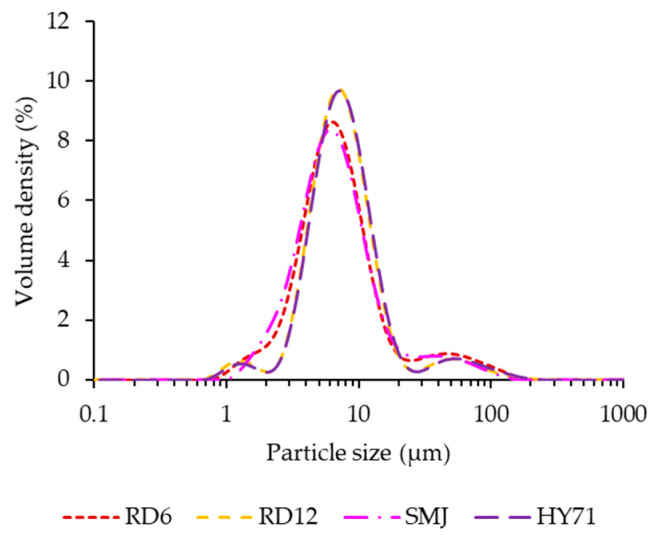
Particle size distribution of waxy starches.

Rice starch granules are classified as small granules (2–10 μm) [36]. The particle size distribution of waxy rice starches determined from light scattering ranged from 3 μm to 23 μm (Figure 1, Table 1). A slightly larger median diameter (D50) of RD12 and HY71 compared to that of RD6 and SMJ (~7.8 μm vs. 6.9 μm, *p* < 0.05) and the former also had a lower value for the polydispersity index (PDI) (1.7 vs. 2.6), indicating differences in granule size and distribution among WRS cultivars. HY71 and RD12 granules were clearly larger, and their size was more uniform, compared to SMJ and RD6. The large granule size of RD12 compared to those of RD6 and SMJ is likely indicative of their characteristics within their respective cultivars, as our results are consistent with previously reported results [5,17]. There were no reports of granule size of HY71. The CLSM images of all WRS confirm the results of the particle size distribution. All granules predominantly displayed an irregular polyhedral shape, which is a botanical characteristic of rice starch granules [3]. 

### 3.2. Chemical Compositions and Molecular Characteristics

The chemical compositions of the WRS were ~12–13% moisture content, 0.08% protein, ~0.01–0.04% fat, and ~0.01–0.03% ash (Table 2). Their low protein and lipid content are due to the absence of waxy gene-granule-bound starch synthase (GBSS) [37]. All waxy rice cultivars contained ~0.71–1.47% amylose (AM) content (*p* > 0.05), determined by the iodine absorptivity method. The values are slightly lower but in line with those reported by Thitisaksakul et al. [18], where their AM content was determined by the precipitation of amylopectin with Concanavalin A.

**Figure 2:**
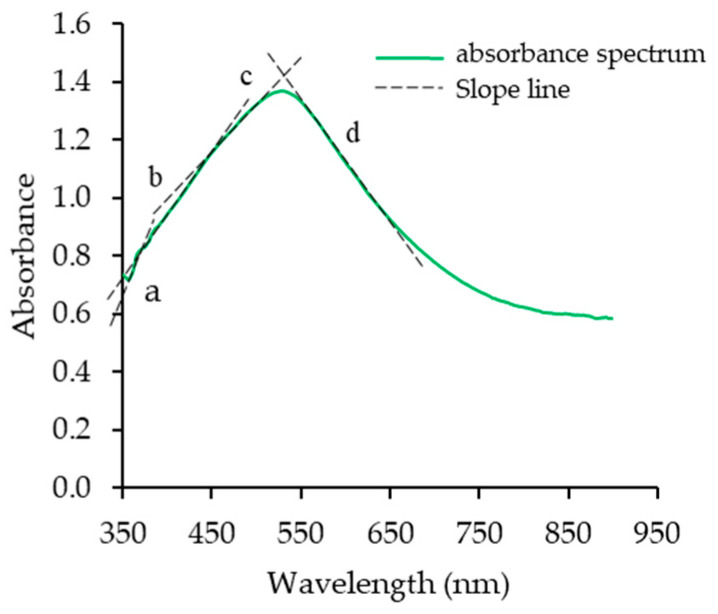
Schematic diagram of spectra of iodine–amylopectin complex and slope region of waxy rice starch. a–d letters indicate the slope positions of each region.

The low iodine absorptivity of WRS, similar to that of Knutson [24], verified amylopectin (AP) was the component responsible for the λ-max absorption (Table 3). The higher λ-max, intrinsic viscosity [η], and $\bar{ECL}$ of RD12 and HY71 (Table 3) suggested they had external long chains, compared to SMJ and RD6. In our study, the ratio of the slope ‘c’ to ‘d’ (Figure 2) of HY71 and SMJ was the highest (0.80) and the lowest (0.69), respectively. The λ_max_ of HY71 and RD12 was higher than RD6 and SMJ (530 nm vs. 528 nm). The results imply there are differences in molecular size and shape of AP. The molecular size of HY71 AP was large, and its external chains were long. On the contrary, the SMJ AP was small and contained shorter branch chains.

Differences in the number–average molecular size of WRS expressed by $\bar{{DP}_{n}}$ were also observed among cultivars. HY71 had the largest molecular size and contained long external chains. RD6, on the contrary, exhibited the smallest molecular size, the longest internal chain length ($\bar{ICL}$), and the shortest $\bar{ECL}$. The average molecular size of SMJ and RD12 was between HY71 and RD6. The shorter chain length ratio of $\bar{ICL}$ to $\bar{ECL}$ of RD12 was consistent with its higher intrinsic viscosity and higher λ-max compared to those of SMJ (38.9% vs. 40%, 112 vs. 107, and 530 nm vs. 528 nm), suggesting that RD12 had longer external chains than its counterpart.

### 3.3. Swelling Characteristics and Thermal Properties of WRS

Effects of AM content and granule shape, which are important factors in the swelling characteristics of WRS, were excluded from this study. When determined in excess water (1% *w*/*w*), HY71, which had the largest $\bar{{DP}_{n}}$ as well as large granules, exhibited the highest swelling power (SP) and a high solubility index (WSI) (Table 4). Its highest gelatinization temperature was in line with its long external chains, implying a high degree of the short-range order of the starch. In contrast, SMJ and RD6 had shorter outer chains as shown by their lower λ-max. The $\bar{ICL}$/$\bar{ECL}$ ratios of SMJ and RD6 were 0.39 and 0.48, respectively, which were also relatively higher than those of RD12 and HY71 (~0.38). Those molecular characteristics normally indicate a lower amount of short-range order, resulting in their lower onset gelatinization temperature (T_o_) and peak gelatinization temperature (T_p_) (Table 3 and Table 4). The results were consistent with the positive correlations between the percentage of DP12–DP22, which reportedly facilitated the formation of short-range order, and the gelatinization temperatures (T_o_, T_p_, and T_c_) [38]. The degree of short-range order was negatively correlated with the granule swelling ability [39]. The subsequent high granule swelling and solubility of SMJ was, thus, thought to be due to their quite large molecular size and their short external chains. In addition, RD12 with its large granule size and long external chains had the least granule swelling and high gelatinization temperature. Seemingly, the effect of particle size was not prominent on the granule swelling in excess water of these WRS, compared to the molecular size and branching characteristics. Kowittaya and Lumdubwong [5] reported a positive correlation between molecular weight and granule swelling capacity. The order of SP, a parameter representing swelling capacity in our study, was consistent with the pattern except RD12. The unusually low granule expansion of that specific waxy starch was likely due to its lipid content and the presence of long external chains. RD12 had the highest lipid content and exhibited the highest λ-max, intrinsic viscosity, and %β-amylolysis, as well as the shortest $\bar{ICL}$/$\bar{ECL}$ ratio, suggesting their highest proportion of long external chains, double helices, and AM-like–lipid complexes.

### 3.4. Rheological Properties of WRS and Granule Behavior

Steeneken [15] demonstrated that gelatinized granules swelled to equilibrium at dilute concentrations. Granules, however, swelled to less than equilibrium when the starch concentration exceeded the close-packing concentration (C*), the concentration at which swollen granules occupy the entire space in a suspension [40]. The C* of all WRS in our study was ~2.29–2.48%, suggesting all waxy granules swelled less than equilibrium at any concentration above the solid content. In the close-packed regime, where the volume fraction of granules (particles) is greater than one, the viscosity of the starch/paste will be determined by granule rigidity and surface interactions [16,41]. We expected the granules in our study to be in the close-packed regime, due to the 10% *w*/*w* starch concentration used in our study, yielding a volume fraction > 1.0 [42]. 

#### 3.4.1. Small Amplitude Oscillatory Shear (SAOS)

Previous studies also examined the effect of types of waxy starch, solid concentration, heating profiles, and applied stress on the stiffness of starch gel/paste. The value of G′ of an 8% native waxy maize starch gel studied by Wang et al. [10] was ~8 Pa, and its tan δ of 0.6 indicated that the material was a soft gel. A 6% WRS gel studied by Boonkor et al. [17] displayed a G′ of ~15 Pa and a tan δ of 0.5. Precha-Atsawanan et al. [9] studied the stiffness of 15% gels from native and debranched WRS, and observed a G′ of 30 Pa for the native starch gel, with a tan δ of 0.3, and a very high G′ of 1000 Pa, with a tan δ of ~0.02 for the debranched starch. They suggested that the soft material prepared from native (granular) waxy starch behaved more like a weak particle gel, while the debranched (non-granular) gel consisted of clusters of short and stiff polymers.

In the dynamic temperature sweep test, the starch suspension was pre-heated to ~4–5 °C below their T_o_ (onset gelatinization temperature, SMJ = 60.5 °C, RD6 = 61.5 °C, RD12 and HY71 = 63.0 °C) to prevent starch settling before loading on the base of the rheometer probe, yielding an initial G′ near 0.1 Pa. A rise in G′ occurred with an increase in temperature ~72–75 °C as starch granules gradually swelled and finally occupied the entire phase volume [43]. The average T_G′max_ of the waxy starches was 76.9–80.8 °C, which was between their individual T_p_ and T_c_ values, indicating G′ of the system continued to increase after the midpoint of gelatinization and ceased before complete gelatinization.

**Figure 3:**
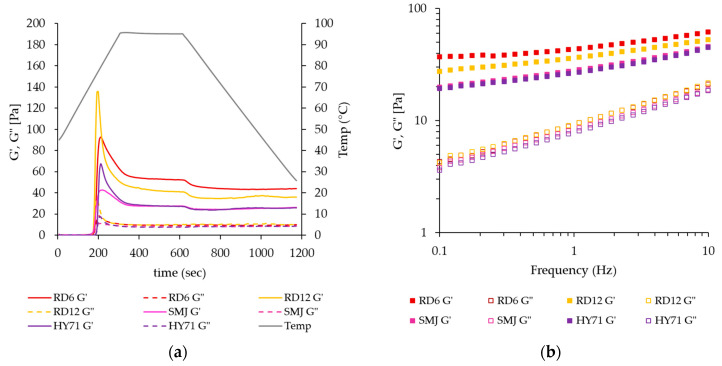
Storage (G′) and loss (G″) moduli during (**a**) temperature sweep tests, and (**b**) frequency sweep tests using small amplitude oscillatory shear measurements. Temperature sweep tests were performed at a strain of 0.1% and frequency of 1 Hz. The frequency sweep was performed at 1% strain, at 25 °C.

The values for G′ and G″ moduli of WRS indicate the system can be considered a soft gel. Differences in G′ among cultivars were observed, while the values for G” of all WRS were quite similar (Figure 3a and Table 5). The G′_max_ of the WRS was the highest for RD12, followed by RD6, HY71, and SMJ having the lowest value. The latter two displayed a lower G′_max_ despite their high swelling ability in the dilute system (Table 4). The results were consistent with previous studies that reported a reverse relationship between gel stiffness and granule swelling capacity in the closed-pack system, except for that of SMJ [15,44]. Sodhi and Singh [45] reported that gels of high AM rice starch with the largest granule size displayed the highest values for G′ and G″. The broad granule size distribution and smaller granule size of RD6 (based on (D[3,2] and D50), whose AP molecules contained short external chains, may have contributed to its lower G′_max_ value than RD12. This could also explain the higher value for this parameter for HY71 compared to SMJ. Long chains may facilitate the formation of new entanglements between branches of AP molecules in swollen granules when heated to 95 °C, subsequently delaying the breakdown of the AP gel matrix in those granules [44].

The decrease in G′ after reaching its peak value was thought to be due to further deformation and disruption of the swollen granules via a breakdown of the AP matrix. Disentanglement of AP chains in swollen granules occurred upon further heating at 95 °C, resulting in granule softening and deformability [44]. A decrease in G’ for larger granule size with narrow distribution occurred to a larger degree as exhibited by RD12 and HY71 vs. RD6 and SMJ. The value of G′ continually decreased during the holding time, but remained relatively stable during the cooling phase.

The 10% native WRS gels from the different cultivars in our study displayed a range of G′ ~20–40 Pa and a range of tan δ_25_ from 0.22 to 0.38, indicating soft gel characteristics as well as validating the significance of granules on waxy gel stiffness. G″_25_ of all WRS was similar (*p* > 0.05). Therefore, differences in tanδ_25_ were due to differences in the G′ of WRS exclusively. At 25 °C, RD6 gel had the highest stiffness followed by RD12, while SMJ and HY71 had lower stiffness with similar G′ (i.e., G′_25_: RD6 > RD12 ≥ SMJ, HY71). The steadily lower G′_25_ compared to G′_max_ of WRS gels implies that molecules of AP leachates between swollen granules could not form strong physical cross-links with each other during the short cooling period, yielding gel stiffness when compared to AM leachates of normal starch [46]. Those leachates were partially interspersed between swollen granules and merely acted as glue. The WRS gels in the concentrated system, thus, consisted of a network of swollen granules tightly packed and partially interspersed with a glue of AP solubles. Granule rigidity and deformability pattern of swollen granules, and their interaction between deformed granules, which was determined by the granule size, particle size distribution, and AP molecular characteristics, predominantly controlled gel stiffness and other solid-like behaviors of the network. The fact that at lower temperature the gel of RD6 was stiffer than RD12 might be because of its high granule rigidity and a high degree of tightly packed granules. The CLSM images of WRS gels at G′_25_ verified our supposition.

The dependence of G′ and G″ on frequency at 25 °C is shown in Figure 3b. When frequency increased, the values for G′ and G″ of the waxy starches increased only moderately. The order of G′ during frequency sweep was the same as for the values of G′_25_ during temperature ramp. The order of gel stiffness was RD6 > RD12 > SMJ, HY71. The power law index of these gels was in a range of 0.11 and 0.18 (see in Supplementary Materials), implying that the gels behave like disordered solids with a very broad relaxation spectrum [47].

#### 3.4.2. Large Amplitude Oscillatory Shear (LAOS)

**Figure 4:**
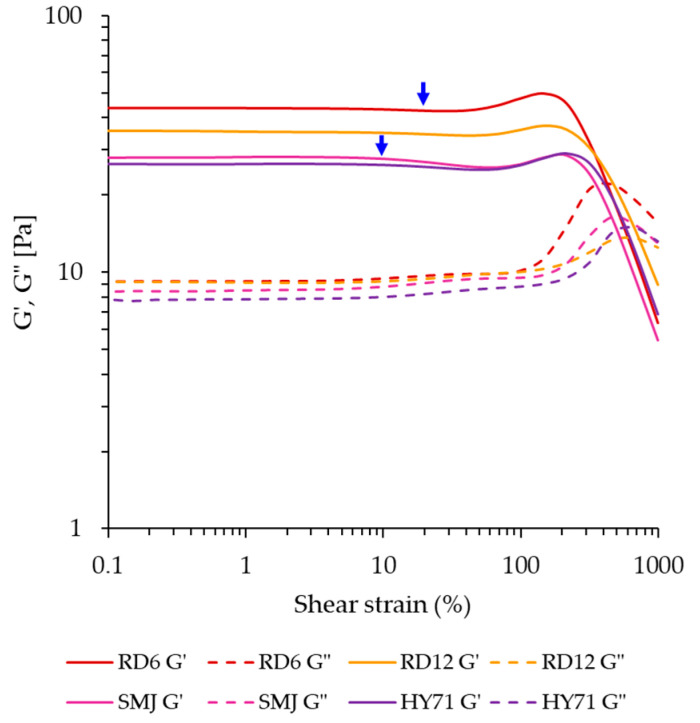
Storage (G′) and loss (G″) moduli of waxy starch gels as a function of shear strain (25 °C, 1 Hz), the arrows indicate the end of the LVE region.

The rheological response of the WRS gels determined by large amplitude oscillatory shear (LAOS) again indicates that RD6 and RD12 were stiffer than SMJ and HY71 (Figure 4). Further, the maximum linear viscoelastic (LVE) strain of the waxy gels where the shear strain of G′ started to change from its constant value in the LVE regime was about 20% strain for RD6 and RD12, and 10% strain for SMJ and HY71 (see arrows in Figure 4). All waxy gels showed an overshoot in both G′ and G″, before decreasing, which was previously classified as strong strain overshoot (type IV) behavior [48,49]. This behavior is observed when the rates of network bond formation and bond disruption are both positive, and in the relevant strain range, bond formation is slightly higher (by a factor between 1 and 2) than the rate of bond disruption. For all gels, the overshoot in G″ (also known as the Payne effect) is more significant than the overshoot in G′. We quantified the former using the Payne height defined in Equation (3).

For a 15% debranched waxy starch gel, Precha-Atsawanan et al. [9] observed a Payne height of ~1.5 at a strain around 1%. The Payne effect of our 10% WRS gels occurred at strains between 100–700% (Figure 4). The variation in deformability of swollen granules acting as fillers in the WRS gels was expected to be the main underlying cause. The peak in G″ of RD6 and SMJ was at a shear strain of ~390% and ~460%, while that of RD12 and HY71 occurred at ~575% and ~685% shear strain.

The Payne height ranged from 0.49 to 1.41, with RD6 (1.41) having the highest value and RD12 having the lowest (0.49). The values for SMJ and HY71 were not significantly different (0.9). In the 6% native (granular) WRS studied by Boonkor et al. [17], the Payne effect was absent, and the material showed type I non-linear behavior. The 15% native WRS studied by Precha-Atsawanan et al. [9] only showed a very mild upswing in G″ around 1000% strain, and this material showed type III behavior. The starch concentration, the applied shear stress, and cooking conditions strongly affect the rigidity of swollen granules, and this appears to have a strong effect on the rheology in the non-linear viscoelastic regime, yielding either type I [17], type III [9], or type IV (this study) behavior.

**Figure 5:**
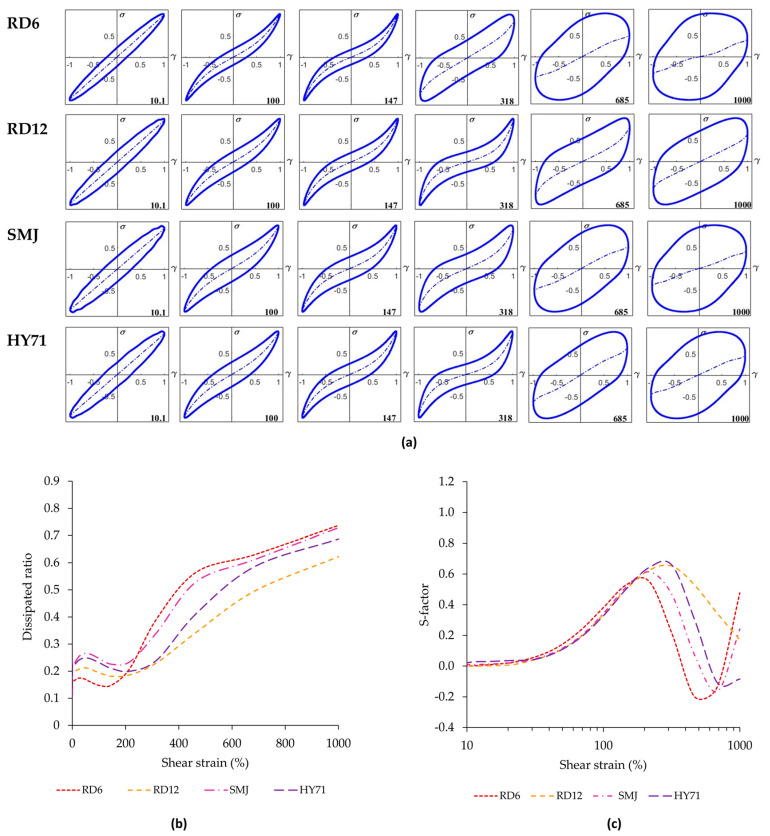
Large amplitude oscillatory shear (**a**) normalized elastic Lissajous plots of stress versus strain (σ (γ)); (**b**) dissipation ratio (calculated with Equation (4)); and (**c**) S-factor of waxy rice gels (calculated with Equation (5)).

The Lissajous curves of all waxy starches at low strain (~10%) had an elliptical shape and differed only slightly with respect to the width of the loop, with RD6 having the narrowest loop (Figure 5a). The width of the loop characterizes the magnitude of the intra-cycle viscous dissipation. At 100% and 147% strain, the loops for all samples displayed an increase in slope towards maximum intra-cycle strain, indicative of strain hardening. In this phase, RD6 still had the narrowest loop, and, hence, the lowest dissipated energy per cycle. SMJ had the widest loop of all samples. At a strain of 318%, the curves for RD12 and HY71 still displayed strain hardening, but the curves of RD6 and SMJ were significantly wider and abruptly changed to a near rhomboidal shape, indicative of significant strain thinning, and, hence, disruption of the gel network. The latter two had nearly circular loops at even higher strains, but the line in the center of the loop, which represents the elastic contribution to the total stress, still had a finite slope. This indicates that there is still residual elasticity present in the gel structure. The RD12 and HY71 samples, which had uniform large granules and long-chain AP molecules, exhibited a more gradual transition from an elliptical shape to a rhomboidal shape. RD6, which contained short-chain AP molecules and had a broad size distribution and smaller granule sizes, exhibited the fastest transition.

The differences in dissipated energy and strain stiffening behavior (S-factor) of waxy starches are shown in Figure 5b,c. At a low shear strain of less than 200%, the dissipation ratio of all WRS gels was ≤0.3, indicating a more elastic-dominated behavior of the materials. The order of the magnitude of the dissipation ratio was RD 6 < RD12 < HY71 < SMJ, which was consistent with their G’_25_ values determined in the SAOS experiments. The behavior of the WRS gels, however, drastically changed to a more plastic behavior after about 200% strain. RD6 showed the steepest increase in dissipation ratio, and beyond 200% strain had the highest value for this parameter. At the highest strain, RD12 had the lowest dissipation ratio, and, hence, the highest level of residual elasticity.

The S-factor of waxy starches was close to zero at 10% strain, and subsequently increased moderately to a maximum value of around 0.6, suggesting all samples showed a similar strain hardening effect, albeit at different intra-cycle strain. The maximum S-factor of RD6 and SMJ (0.55–0.61) at ~200% strain was slightly lower than that of RD12 and HY71 (0.65) at ~300% strain. This is most likely linked to the fact that RD12 and HY71 had longer exterior chain APs, whereas RD6 and SMJ had APs with shorter exterior chains. This observation is consistent with the higher S-factor of the 15% debranched WRS gel compared to that of native WRS gel (3 vs. 0.2) previously studied by Precha-Atsawanan et al. [9]. After reaching a maximum in the S-factor, RD6, SMJ, and HY71 showed a more abrupt decrease in the S-factor, to negative values, indicative of strain softening, and, hence, disruption of the network structure. RD12 showed a more gradual network breakdown. Seemingly, the presence of long-chain amylopectin increased strain stiffening and delayed breakdown. The increase in S-factor above 500% strain is apparent strain hardening and not real strain hardening. For perfectly plastic behavior, the Lissajous curve takes on the shape of a rectangle, with G′M → 0, and, hence, S → 1, according to Equation (5). The overall behavior of the samples at these high strains is still strain softening.

#### 3.4.3. Microstructure of Granules and Pastes/Gels at Low Shear

**Figure 6:**
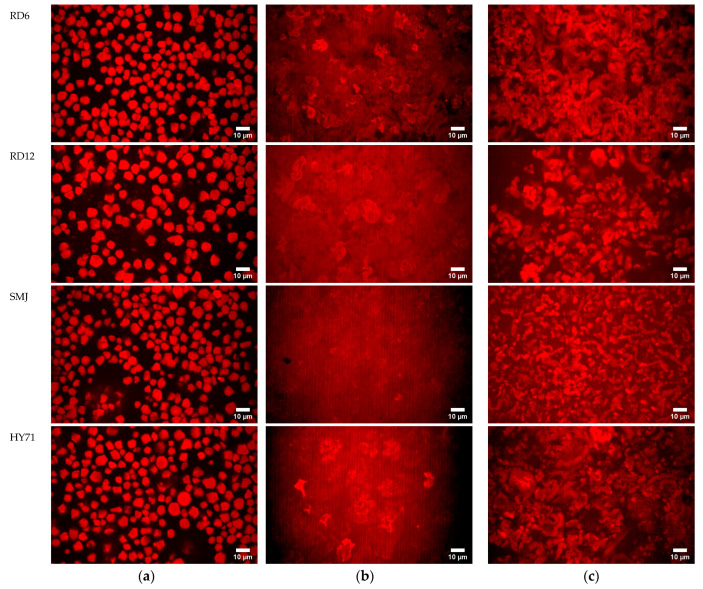
Microstructure of (**a**) waxy starches before heating; (**b**) waxy pastes/gels at the point where G′ = G′_max_; and (**c**) at the point where G′= G′_25,_ during the temperature sweep, imaged with CSLM at a magnification of 60×; scale bar in the images equals 10 μm.

The microstructure of WRS granules, pastes, and/or gels during the temperature ramp was observed using CLSM (Figure 6). Nile blue reagent containing water-soluble basic oxazine was used as a fluorescent dye to observe native starch granules [50]. This selective dye was also used to investigate the structures of waxy and non-waxy starch (1% starch) during gelatinization. In a preliminary experiment, in images of 10% WRS gels after heating and cooling (at G′_25_), stained with Nile blue dye, granules were obscured by molecules that leached out. Therefore, removal of the soluble phase and dilution were applied to observe granule characteristics.

The CLSM images of the gelatinized waxy samples at the point where G′ = G′_max_ in the temperature sweep (Figure 6b) displayed deformed granules, and AP leachates (solubles), the latter being evident from the (near) uniform red background. The edges of the deformed swollen granules could still clearly and visibly be observed in the gel of RD12 and RD6, and the size of the distorted granules of RD12 was larger than that of RD6. Large swollen and deformed granules of HY71 were also still noticeable within the red dense background, confirming a high amount of AP leachates. SMJ displayed mostly a dense background and its deformed granules were scarcely visible, consistent with its highest level of molecule leaching.

The CLSM images of the stained sediment of 10% *w*/*w* gelatinized WRS at G′_25_ show that their microstructure resembles that of aggregated particle gels, embedded in a continuous phase with leached AP. However, there were significant differences in structure between samples. RD6 showed a fine-meshed more strand-like gel with small and uniformly distributed voids. RD12 formed a much more heterogeneous gel than RD6, with larger and more spherical clusters and larger voids. The voids had a more intense red color than RD6 gels, probably because of the higher level of leachates. SMJ is again a more fine-stranded gel, with strands longer than RD6, but also contains more voids, with a more intense red background resulting from the leachates. Finally, HY71 is again a very coarse gel, consisting of large clusters and large voids.

The combination of small granule size, a large particle size distribution, low-molecular-weight amylopectin (AP) with short external chains, and high lipid content imparted high granule rigidity. It facilitated the formation of a fine-meshed, more strand-like particle waxy gel structure interspersed by small, uniformly distributed voids. Larger voids and more AP leachates were observed when the waxy starch had large molecular weight AP and low lipid content. On the contrary, a combination of large granule size, a narrow particle size distribution, high-molecular-weight AP with long external chains, and high lipid content contributed to a lesser granule rigidity. Deformed granules clustered and a more heterogeneous gel was formed. The gel structure became increasingly heterogeneous and coarse as the molecular size of the amylopectin (AP) increased, particularly when the starch contained a lower lipid content.

RD6, which had the most homogeneous and finely stranded gel, also had the highest value for G′_25_. It also had slightly more strain hardening and a higher Payne height in the non-linear regime, and it yielded more abruptly, leading to a higher dissipation ratio at strains over 100%. RD12 had a lower value for G′_25_, the lowest Payne height, and displayed much more gradual softening and the lowest dissipation ratio at high strain. This sample had a low SP, high level of leachates, and the AP had long exterior chains. This could mean its rheology is determined mostly by the entangled AP background and its more deformed granules. SMJ also has significant leaching of AP, but shorter external chains, leading to a weaker gel, compared to RD12.

### 3.5. Pasting Properties

**Figure 7:**
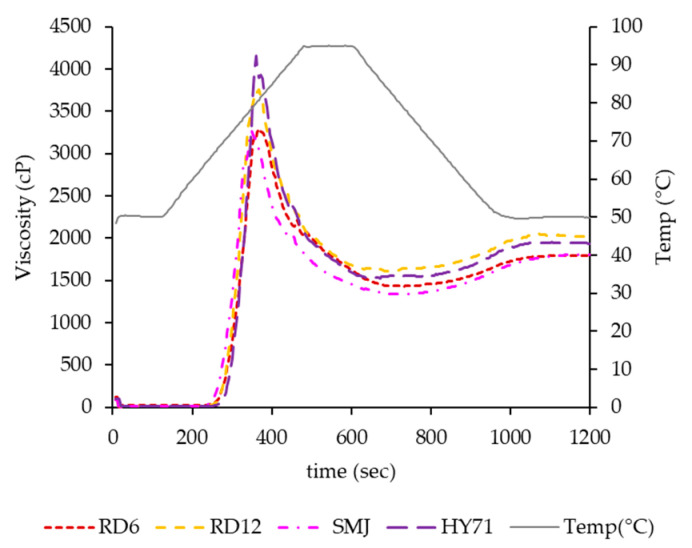
Pasting properties of waxy rice starches.

**Figure 8:**
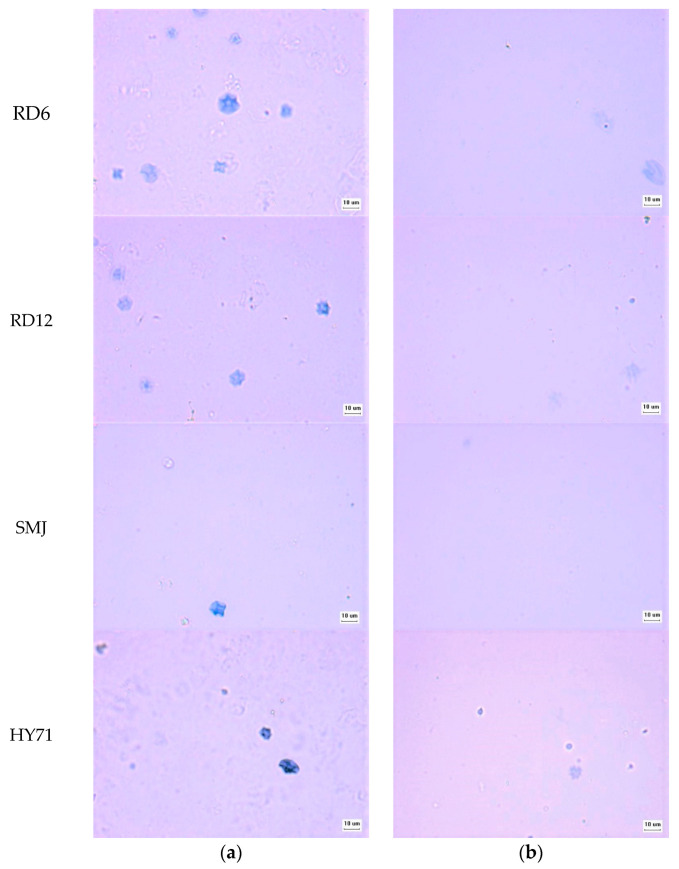
Microstructure of waxy pastes at (**a**) PV; and (**b**) FV with light microscope at a magnification of 40×; scale bar equals 10 μm.

Pasting properties of the WRS samples were different, as shown in Figure 7 and Table 6, and pasting temperature (PT) of waxy starches followed the order of their T_o_ values (Table 4). The degree of deformability of swollen granules was also greatly affected by the applied shear stress [41]. It was, therefore, not unexpected that pasting profiles of all waxy rice starches, especially the peak viscosity (PV) was not consistent with their G′_max_ value. The PV of HY71 was the highest, followed by that of RD12, RD6, and SMJ, indicating large granule size with a uniform distribution provided high peak viscosity. A similar finding was reported by Singh et al. [51], where positive correlations of pasting properties (PV, BD, FV, and SB) and granule size of wheat starches were found. The high surface volume of HY71 and RD12 expressed by their D[3,2] might have facilitated water diffusion into granules. Consequently, their heating slope was significantly higher than that of RD6 and SMJ. The large molecular size of HY71 was another factor magnifying the rise of PV, as previously reported [5]. The lower PV of RD6 and SMJ was thought to be due to their smaller-size granules with a wide particle size distribution (high PDI). The very low granule rigidity of SMJ, consistent with its low G′_max_ and its CLSM image of G′_25_ was also evident from the almost absence of iodine-stained granules at the PV and its lowest hot paste viscosity (HV) (Figure 7 and Table 6). Granule remnants of HY71, RD6, and RD12 at their PV were shown; the remnant size of HY71 was large whereas discrete clusters of swollen granules of RD6 were observed (Figure 8). This confirmed the trends of granule deformability of those waxy starches in the SAOS and LAOS experiments. The FV of the waxy starches in this study was similar, insinuating a complete disruption of waxy granules. Their differences in molecular characteristics were not substantial enough to affect their FV.

### 3.6. Findings of the Study and Evaluating the Applicability of Waxy Rice Starches for Food Application

The SAOS and LAOS rheological behaviors of 10%WRS gels from four cultivars confirmed differences in their gel characteristics. Differences in the texture of cohesive waxy starch gels were suggested to be primarily influenced by the varying degrees of deformability and rigidity of starch granules. Those parameters were further attributed to a combination of factors, including granule size, particle size distribution, molecular size, external chain length of amylopectin, and lipid content. The findings can be applied to select waxy rice starches that are appropriate for the desirable texture of food products and food processing. With the ability to form a soft and weak gel including its pasting properties, and low PV, HV, FV, BD, and SB, the SMJ waxy rice starch would be an excellent option for Chinese sticky rice dumplings, commonly known as Zong Zi. Due to its ability to form a stiff yet weak gel and its paste properties, which are resistant to shear and low degree of retrogradation, RD6 would be a good choice for puddings and low-fat yoghurt. RD12 and HY71 imparted stronger gels (longer strain), which would be suitable for use as meat-binding agents, allowing for tailoring textures with varying degrees of stiffness.

## 4. Conclusions

The microstructure and rheological properties of 10% native WRS gels confirmed that they were aggregated particle gels within a continuous phase of leached AP. The rheological properties of waxy gels were impacted by variations in deformability and rigidity of granules, which were affected by granule size and distribution, molecular size, the external chain lengths of AP, and lipid content. The ability of the granules to swell and the concentration of AP leachates governed the G′_max_ under SAOS. Larger granules with a uniform distribution tended to demonstrate higher G′_max_ values when comparing the waxy gels with similar swelling ability and leaching. The gel stiffness expressed by G′_25_ was related to granule rigidity and AP solubles. The WRS gels with different granule deformability exhibited distinct non-linear rheological responses under LAOS. Stiff gels formed by RD6 showed a more abrupt change from elastic to plastic behavior. Waxy gels with long exterior chains displayed a slower transition. The Payne effect was also observed, and the effect was more prominent in the starches with rigid granules. The findings provide insights for the food industry for the selection of waxy rice starches suitable for their end-product characteristics. We suggest investigating effects of concentrations, shear forces, and storage temperature on LAOS behaviors of waxy rice starch gels and the texture of starch-based foods for future research. 

## Figures and Tables

**Table 1:** Particle size distribution of waxy starches.

Cultivar	Granule Size (μm)			D[3,2]	PDI
	D10	D50	D90		
RD6	3.20 ± 0.01 ^c^	7.07 ± 0.04 ^b^	23.00 ± 1.41 ^a^	5.82 ± 0.04 ^c^	2.80 ± 0.19 ^a^
RD12	3.96 ± 0.02 ^b^	7.76 ± 0.16 ^a^	17.10 ± 1.70 ^b^	6.34 ± 0.09 ^b^	1.69 ± 0.18 ^b^
SMJ	3.07 ± 0.01 ^d^	6.84 ± 0.16 ^b^	19.35 ± 1.77 ^ab^	5.76 ± 0.08 ^c^	2.38 ± 0.20 ^a^
HY71	4.07 ± 0.01 ^a^	7.95 ± 0.08 ^a^	17.75 ± 1.20 ^b^	6.59 ± 0.05 ^a^	1.72 ± 0.13 ^b^

Mean values in the same column with different letters are significantly different (*p* < 0.05).

**Table 2:** Chemical compositions of waxy starches.

Cultivar	Moisture Content (%)	Protein (%)	Fat (%)	Ash (%)	AM (%)
RD6	12.20 ± 0.01 ^c^	0.08 ± 0.00 ^a^	0.02 ± 0.01 ^a^	0.01 ± 0.00 ^c^	1.47 ± 0.51 ^a^
RD12	13.51 ± 0.11 ^a^	0.08 ± 0.00 ^a^	0.04 ± 0.02 ^a^	0.03 ± 0.00 ^a^	0.71 ± 0.05 ^a^
SMJ	12.28 ± 0.04 ^c^	0.08 ± 0.00 ^a^	0.01 ± 0.00 ^a^	0.02 ± 0.00 ^b^	1.03 ± 0.05 ^a^
HY71	12.60 ± 0.06 ^b^	0.08 ± 0.00 ^a^	0.03 ± 0.01 ^a^	0.02 ± 0.00 ^b^	1.26 ± 0.92 ^a^

Mean values in the same column with different letters are significantly different (*p* < 0.05).

**Table 3:** Spectrum parameters, intrinsic viscosity, $\bar{{DP}_{n}}$, and branch chain profiles of waxy starches.

Cultivar	Absorbance Profiles			[η]	$\bar{{DP}_{n}}$	Branch Chain Profiles				
	λ-max (nm)	Absorptivity	Ratio Slope c/d			%β	$\bar{NC}$	$\bar{CL}$ (AGU)	$\bar{ECL}$ (AGU)	$\bar{ICL}$ (AGU)
RD6	528.5 ± 0.7 ^b^	1.37 ± 0.12 ^a^	0.745 ± 0.010 ^b^	102.73 ± 2.99 ^b^	2209 ± 189 ^b^	54.16 ± 0.29 ^b^	136 ± 2 ^b^	20.19 ± 0.92 ^a^	12.93 ± 0.06 ^d^	6.25 ± 0.06 ^a^
RD12	530.0 ± 0.0 ^a^	1.13 ± 0.04 ^a^	0.744 ± 0.025 ^b^	112.33 ± 2.04 ^a^	2758 ± 212 ^ab^	58.03 ± 0.64 ^a^	190 ± 19 ^a^	19.46 ± 1.79 ^a^	13.29 ± 0.86 ^c^	5.17 ± 0.62 ^c^
SMJ	528.0 ± 0.0 ^b^	1.24 ± 0.28 ^a^	0.696 ± 0.009 ^c^	105.64 ± 0.83 ^ab^	2759 ± 279 ^ab^	58.35 ± 0.61 ^a^	154 ± 10 ^ab^	20.44 ± 0.97 ^a^	13.93 ± 0.47 ^b^	5.51 ± 0.35 ^b^
HY71	530.0 ± 0.0 ^a^	1.35 ± 0.73 ^a^	0.802 ± 0.008 ^a^	107.62 ± 3.30 ^ab^	3362 ± 364 ^a^	59.01 ± 0.08 ^a^	173 ± 22 ^ab^	20.98 ± 1.53 ^a^	14.38 ± 0.74 ^a^	5.60 ± 0.51 ^b^

Mean values in the same column with different letters are significantly different (*p* < 0.05).

**Table 4:** Swelling characteristics and thermal properties of waxy rice starches.

**Cultivar**	**Swelling Properties**			**Gelatinization Properties**				
	**SP**	**WSI (%)**	**ϕ_10%_**	**T_o_ (°C)**	**T_p_ (°C)**	**T_c_ (°C)**	**T_c_–T_o_ (°C)**	**ΔH (J/g)**
RD6	54.76 ± 1.70 ^bc^	21.44 ± 1.60 ^b^	4.30 ± 0.11 ^ab^	65.97 ± 0.33 ^b^	74.58 ± 0.09 ^ab^	86.21 ± 1.51 ^a^	20.24 ± 1.84 ^a^	6.28 ± 2.05 ^a^
RD12	52.92 ± 1.26 ^c^	23.72 ± 3.60 ^ab^	4.03 ± 0.09 ^c^	66.68 ± 0.33 ^ab^	74.85 ± 0.43 ^a^	86.39 ± 0.12 ^a^	19.71 ± 0.21 ^a^	6.91 ± 0.92 ^a^
SMJ	57.12 ± 1.14 ^b^	29.10 ± 0.01 ^a^	4.05 ± 0.08 ^bc^	64.84 ± 0.29 ^b^	73.73 ± 0.33 ^b^	84.61 ± 0.45 ^a^	19.77 ± 0.16 ^a^	7.16 ± 0.13 ^a^
HY71	60.71 ± 0.26 ^a^	28.02 ± 1.70 ^a^	4.37 ± 0.08 ^a^	67.15 ± 0.23 ^a^	75.49 ± 0.46 ^a^	86.74 ± 0.68 ^a^	19.60 ± 0.45 ^b^	6.83 ± 0.51 ^a^

Mean values in the same column with different letters are significantly different (*p* < 0.05).

**Table 5:** Rheological properties of waxy starches during temperature ramp and amplitude sweep test.

**Parameters**	**RD6**	**RD12**	**SMJ**	**HY71**
Temperature sweep test				
G′_max_ (Pa)	93.14 ± 17.52 ^b^	138.45 ± 4.96 ^a^	44.64 ± 18.39 ^c^	67.28 ± 13.42 ^bc^
G″_max_ (Pa)	18.14 ± 4.51 ^b^	34.30 ± 0.53 ^a^	11.84 ± 4.43 ^b^	17.43 ± 3.29 ^b^
T_o G′_ (°C)	72.72 ± 0.78 ^b^	72.31 ± 0.04 ^b^	71.74 ± 0.81 ^b^	75.27 ± 0.14 ^a^
T_G′max_ (°C)	79.81 ± 0.78 ^a^	76.85 ± 0.76 ^a^	80.83 ± 3.46 ^a^	79.82 ± 0.86 ^a^
tan δ_G′max_	0.18 ± 0.01 ^b^	0.24 ± 0.01 ^a^	0.25 ± 0.00 ^a^	0.25 ± 0.01 ^a^
G′_25_ (Pa)	44.14 ± 0.48 ^a^	35.88 ± 0.69 ^b^	26.13 ± 2.79 ^c^	25.89 ± 1.10 ^c^
G″_25_ (Pa)	9.78 ± 0.18 ^a^	10.07 ± 0.16 ^a^	8.76 ± 1.19 ^a^	8.52 ± 0.20 ^a^
tan δ_25_	0.22 ± 0.01 ^c^	0.28 ± 0.00 ^b^	0.34 ± 0.01 ^a^	0.33 ± 0.01 ^a^
Amplitude sweep test				
G′_LVE_ (Pa)	43.56 ± 1.67 ^a^	35.20 ± 1.10 ^b^	28.19 ± 2.81 ^c^	26.39 ± 0.19 ^c^
G″_LVE_ (Pa)	9.25 ± 0.38 ^a^	9.13 ± 0.21 ^a^	8.49 ± 0.89 ^a^	7.86 ± 0.06 ^a^
tanδ_1%_	0.21 ± 0.02 ^c^	0.26 ± 0.00 ^b^	0.30 ± 0.00 ^a^	0.30 ± 0.00 ^a^
G″_max_ (Pa)	22.31 ± 0.89 ^a^	13.64 ± 0.63 ^b^	16.35 ± 2.41 ^b^	14.83 ± 1.52 ^b^
Payne height	1.41 ± 0.00 ^a^	0.49 ± 0.03 ^c^	0.92 ± 0.08 ^b^	0.89 ± 0.18 ^b^

Mean values in the same row with different letters are significantly different (*p* < 0.05).

**Table 6:** Pasting properties of waxy rice starches.

Cultivar	PT (°C)	Peak Time (min)	Viscosity (cP)					Heating Slope	BD Slope
			PV	HV	BD	FV	SB		
RD6	67.48 ± 0.04 ^b^	6.27 ± 0.19 ^a^	3454 ± 228 ^bc^	1511 ± 113 ^a^	1943 ± 115 ^c^	1879 ± 127 ^a^	368 ± 13 ^b^	37.9 ± 1.4 ^c^	−5.83 ± 0.15 ^c^
RD12	67.43 ± 0.04 ^b^	6.13 ± 0.00 ^a^	3753 ± 8 ^b^	1582 ± 35 ^a^	2171 ± 27 ^b^	1973 ± 64 ^a^	391 ± 30 ^b^	45.5 ± 0.0 ^b^	−7.13 ± 0.06 ^b^
SMJ	65.90 ± 0.07 ^c^	5.80 ± 0.00 ^b^	3230 ± 53 ^c^	1330 ± 11 ^b^	1900 ± 42 ^c^	1793 ± 23 ^a^	463 ± 13 ^a^	39.7 ± 0.3 ^c^	−5.93 ± 0.12 ^c^
HY71	68.88 ± 0.04 ^a^	6.00 ± 0.00 ^ab^	4155 ± 4 ^a^	1524 ± 7 ^a^	2631 ± 11 ^a^	1948 ± 12 ^a^	424 ± 19 ^ab^	57.5 ± 0.4 ^a^	−9.59 ± 0.20 ^a^

Mean values in the same column with different letters are significantly different (*p* < 0.05). Slopes were analyzed by differential values of viscosity/time with Origin software.

## Data Availability

The original contributions presented in the study are included in the article/Supplementary Materials, further inquiries can be directed to the corresponding author.

## Supplementary Materials

The following supporting information can be downloaded at: https://www.mdpi.com/article/10.3390/foods13121864/s1, Table S1: Raw data and calculated data of particle size of waxy rice starches determined with Mastersizer; Table S2: Raw data and calculation of ratio slope of absorbance profile; Table S3: Raw data and calculation of β-amylolysis; Table S4: Raw data and calculation for intrinsic viscosity of RD6; Figure S1: Absorbance profile of RD6 starch calculated slopes with Origin software; Figure S2: Standard curves of maltose and glucose; Figure S3: Reduced viscosity versus concentration curve; Figure S4: Power law index of G’ during frequency sweep of waxy rice gels.
