# Supplementary material for: Impact of Deformability and Rigidity of Starch Granules on Linear and Non-Linear Rheological Behavior of Waxy Rice Starch Gels and Applicability for Food End Uses
Source: Foods. 2024 Jun 13;13(12):1864. doi: 10.3390/foods13121864 (PMC11202675; doi:10.3390/foods13121864)
Supplement: Supplementary file 1 [file foods-13-01864-s001.zip › foods-3028929-supplementary.pdf]

# Supplementary materials

## Particle size and distribution

**Table S1** Raw data and calculated data of particle size of waxy rice starches determined with Mastersizer.

| Record No. | Sample Name | Specific Surface Area | Laser Obscuration | D [4,3] | D [3,2] | Dx (10) | Dx (50) | Dx (90) | PDI |
|---|---|---|---|---|---|---|---|---|---|
| 5 | r6-U2 | 976.3 | 7.5 | 13.2 | 5.85 | 3.21 | 7.1 | 25.1 | |
| 6 | r6-U2 | 977.7 | 7.47 | 13 | 5.84 | 3.2 | 7.09 | 24.4 | |
| 7 | r6-U2 | 980.7 | 7.44 | 12.5 | 5.83 | 3.2 | 7.08 | 22.9 | |
| 8 | Average of 'r6-U2' | 978.3 | 7.47 | 12.9 | 5.84 | 3.2 | 7.09 | 24 | 2.93 |
| 9 | r6-U3 | 989.3 | 8.15 | 12.1 | 5.78 | 3.18 | 7.02 | 21.1 | |
| 10 | r6-tU3 | 989.9 | 8.12 | 12.3 | 5.77 | 3.18 | 7.02 | 20.8 | |
| 11 | r6-U3 | 979.3 | 8.09 | 13.2 | 5.84 | 3.2 | 7.09 | 24.3 | |
| 12 | Average of 'r6-U3' | 986.2 | 8.12 | 12.5 | 5.79 | 3.19 | 7.04 | 22 | 2.67 |
| 17 | Smj-ur1 | 1004 | 6.4 | 10.5 | 5.69 | 3.06 | 6.72 | 17.9 | |
| 18 | Smj-ur1 | 1003 | 6.39 | 10.5 | 5.7 | 3.06 | 6.73 | 17.9 | |
| 19 | Smj-ur1 | 998.7 | 6.36 | 10.8 | 5.72 | 3.07 | 6.75 | 18.5 | |
| 20 | Average of 'Smj-ur1' | 1002 | 6.39 | 10.6 | 5.7 | 3.06 | 6.73 | 18.1 | 2.23 |
| 21 | Smj-ur2 | 984.1 | 8.54 | 11.5 | 5.81 | 3.07 | 6.94 | 20.3 | |
| 22 | Smj-ur2 | 983.3 | 8.51 | 11.7 | 5.81 | 3.07 | 6.95 | 20.5 | |
| 23 | Smj-ur2 | 981 | 8.49 | 11.8 | 5.82 | 3.07 | 6.96 | 20.9 | |
| 24 | Average of 'Smj-ur2' | 982.8 | 8.51 | 11.7 | 5.81 | 3.07 | 6.95 | 20.6 | 2.52 |
| 41 | W2-r12-ur1 | 888.3 | 8.98 | 13.8 | 6.43 | 3.98 | 7.9 | 18.9 | |
| 42 | W2-r12-ur1 | 899.1 | 8.95 | 12 | 6.36 | 3.96 | 7.83 | 17.6 | |
| 43 | W2-r12-ur1 | 889.8 | 8.94 | 13.5 | 6.42 | 3.98 | 7.89 | 18.6 | |
| 44 | Average of 'W2-r12-ur1' | 892.4 | 8.96 | 13.1 | 6.4 | 3.97 | 7.87 | 18.3 | 1.82 |
| 45 | W2-r12-ur2 | 911.6 | 6 | 11.1 | 6.27 | 3.94 | 7.65 | 16 | |
| 46 | W2-r12-ur2 | 911.4 | 5.99 | 11.1 | 6.27 | 3.94 | 7.66 | 16 | |
| 47 | W2-r12-ur2 | 913 | 5.98 | 10.8 | 6.26 | 3.94 | 7.65 | 15.8 | |
| 48 | Average of 'W2-r12-ur2' | 912 | 5.99 | 11 | 6.27 | 3.94 | 7.65 | 15.9 | 1.56 |
| 49 | W4-Hy-ur1 | 875.4 | 6.77 | 11.6 | 6.53 | 4.06 | 7.87 | 16.6 | |
| 50 | W4-Hy-ur1 | 872.1 | 6.76 | 12 | 6.55 | 4.06 | 7.89 | 17 | |
| 51 | W4-Hy-ur1 | 871.1 | 6.75 | 11.8 | 6.56 | 4.07 | 7.9 | 17.1 | |
| 52 | Average of 'W4-Hy-ur1' | 872.8 | 6.76 | 11.8 | 6.55 | 4.06 | 7.89 | 16.9 | 1.63 |
| 53 | W4-Hy-ur2 | 868.5 | 8.29 | 12.6 | 6.58 | 4.06 | 7.97 | 18.1 | |
| 54 | W4-Hy-ur2 | 857.8 | 8.27 | 14.9 | 6.66 | 4.07 | 8.04 | 19.6 | |
| 55 | W4-Hy-ur2 | 863.7 | 8.25 | 13.7 | 6.62 | 4.07 | 8 | 18.6 | |
| 56 | Average of 'W4-Hy-ur2' | 863.3 | 8.27 | 13.7 | 6.62 | 4.07 | 8 | 18.6 | 1.82 |

- Average data of each measurement was used to calculate mean values of granule size.
- Polydispersity index (PDI) is calculated as

$$\text{PDI} = \frac{\text{D90} - \text{D10}}{\text{D50}}$$

For example,

$$\text{PDI of RD6} = \frac{24 - 3.2}{7.09} = 2.93$$

**Absorbance profile**

- Maximum wavelength (λ-max) and linear slope 'c' and 'd' of absorbance profile were calculated using Origin software.

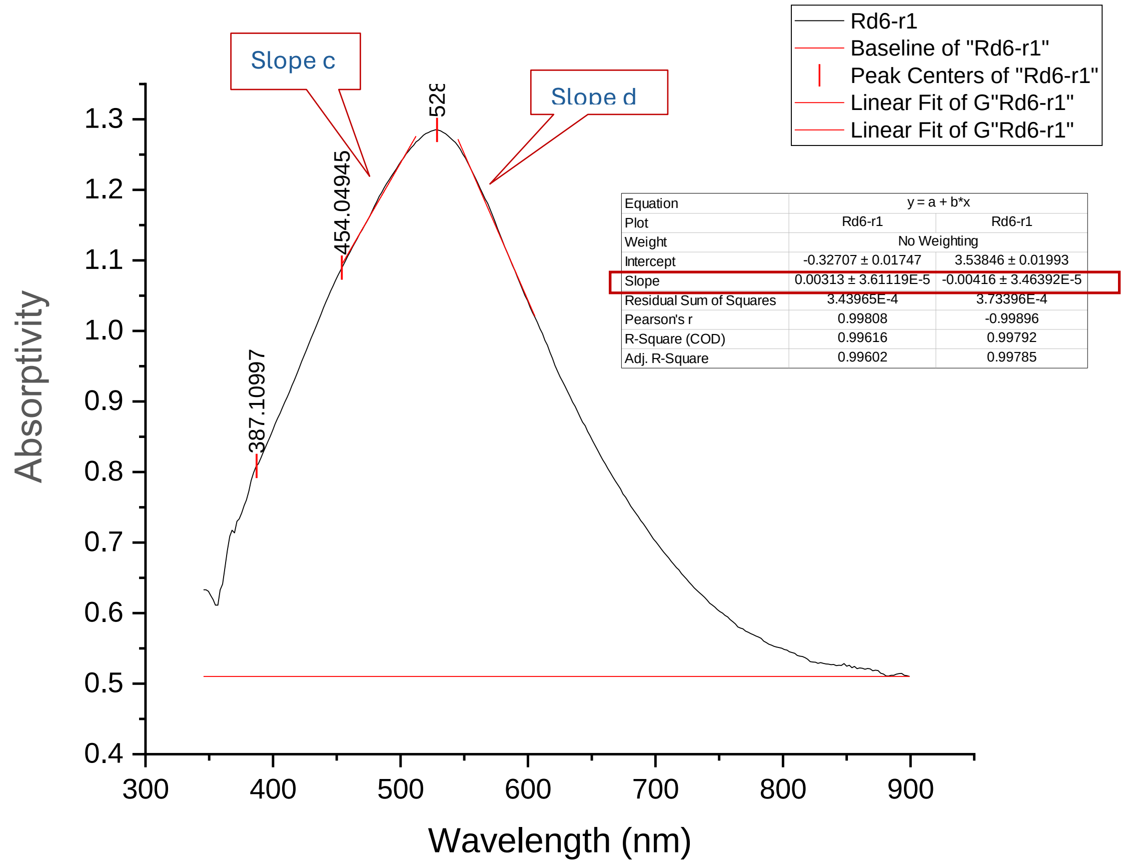


**Figure S1.** Absorbance profile of RD6 starch calculated slopes with Origin software.

**Table S2** Raw data and calculation of ratio slope of absorbance profile

| sample | rep | λ-max | avg | SD | Absorptivity | avg | SD | Slope -c | Slope-d | ratio slope c/d | avg | SD |
|---|---|---|---|---|---|---|---|---|---|---|---|---|
| RD6 | 1 | 528 | 528.5 | 0.7 | 1.29 | 1.37 | 0.12 | 0.00313 | -0.00416 | -0.752 | -0.745 | 0.010 |
| | 2 | 529 | | | 1.45 | | | 0.00310 | -0.00420 | -0.738 | | |
| RD12 | 1 | 530 | 530.0 | 0.0 | 1.10 | 1.13 | 0.04 | 0.00279 | -0.00384 | -0.727 | -0.744 | 0.025 |
| | 2 | 530 | | | 1.16 | | | 0.00297 | -0.00390 | -0.762 | | |
| SMJ | 1 | 528 | 528.0 | 0.0 | 1.04 | 1.24 | 0.28 | 0.00255 | -0.00370 | -0.689 | -0.696 | 0.009 |
| | 2 | 528 | | | 1.44 | | | 0.00283 | -0.00403 | -0.702 | | |
| HY71 | 1 | 530 | 530.0 | 0.0 | 0.83 | 1.35 | 0.73 | 0.00298 | -0.00369 | -0.808 | -0.802 | 0.008 |
| | 2 | 530 | | | 1.86 | | | 0.00457 | -0.00574 | -0.796 | | |

**β-amylolysis**

- Amount of maltose released, and total carbohydrate of sample are calculated by using maltose standard equation, and glucose standard equation, respectively.
- β-amylolysis is calculated by;

$$\beta - amylolysis\ (\%) = \frac{wt.of\ maltose\ released}{wt.of\ maltose\ expressed\ by\ starch}\ x\ 100$$

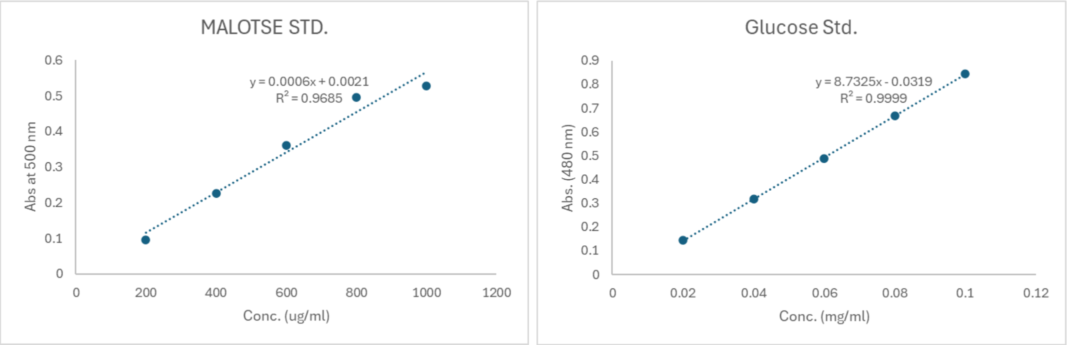


**Figure S2.** Standard curves of maltose and glucose.

**Table S3** Raw data and calculation of β-amylolysis

| | y = 0.0006x + 0.0021 | | | | | y = 8.7325x - 0.0319 | | | | | | | |
|---|---|---|---|---|---|---|---|---|---|---|---|---|---|
| | maltose released | | | | | Total CHO | | | | | % β-amylolysis | | |
| Sample | Abs. (500nm) | ug/0.5ml | mg/ml | mg/324 | | Abs. (480nm) | Glu (dil. 1:25) | Glu (mg/ml) | starch wt. | starch wt. /324 | | avg | SD |
| RD6 | 0.352 | 583.17 | 1.17 | 0.003600 | | 0.807 | 0.0961 | 2.402 | 2.161 | 0.006671 | 53.96 | | |
| | 0.358 | 593.17 | 1.19 | 0.003662 | | 0.815 | 0.0970 | 2.425 | 2.182 | 0.006735 | 54.37 | 54.16 | 0.29 |
| RD12 | 0.342 | 566.50 | 1.13 | 0.003497 | | 0.720 | 0.0861 | 2.153 | 1.937 | 0.005979 | 58.48 | | |
| | 0.339 | 561.50 | 1.12 | 0.003466 | | 0.758 | 0.0905 | 2.261 | 2.035 | 0.006282 | 55.18 | 56.83 | 2.34 |
| SMJ | 0.388 | 643.17 | 1.29 | 0.003970 | | 0.830 | 0.0987 | 2.468 | 2.221 | 0.006854 | 57.92 | | |
| | 0.421 | 698.17 | 1.40 | 0.004310 | | 0.890 | 0.1056 | 2.639 | 2.375 | 0.007331 | 58.78 | 58.35 | 0.61 |
| HY71 | 0.376 | 623.17 | 1.25 | 0.003847 | | 0.787 | 0.0938 | 2.344 | 2.110 | 0.006512 | 59.07 | | |
| | 0.388 | 643.17 | 1.29 | 0.003970 | | 0.815 | 0.0970 | 2.425 | 2.182 | 0.006735 | 58.95 | 59.01 | 0.08 |

Note : molecular weight of maltose = 324

Fore example;

$$\beta - amylolysis\ (\%) = \frac{0.003600}{0.006671}\ x\ 100\ \ = 53.96\ \%$$

## Intrinsic viscosity

The Intrinsic viscosity calculated by extrapolation at zero concentration follows from Huggins's equation:

$$[\eta] = \lim_{c \to 0} \frac{\eta_{sp}}{C}$$

$$\frac{\eta_{sp}}{C} = [\eta] + k'[\eta]^2 C$$

where;

k' = Huggins's constant, C = concentration

Specific viscosity $(\eta_{sp}) = \eta_{rel} - 1$

Relative viscosity $(\eta_{rel}) = \frac{t}{t_0}$

t = efflux time for solution, $t_0$ = efflux time for solvent

**Table S4** Raw data and calculation for intrinsic viscosity of RD6

**RD6**

| Conc. (g/ml) | dup | time (sec) | avg. time (sec) | relative viscosity ($\eta_{rel}$) | specific viscosity ($\eta_{sp}$) | reduce visco ($\eta_{red} = \eta_{sp}/C$) |
|---|---|---|---|---|---|---|
| 0.0030 | 1 | 233.97 | 234.10 | 1.40 | 0.40 | 133.78 |
| | 2 | 234.22 | | | | |
| 0.00240 | 1 | 214.63 | 215.24 | 1.29 | 0.29 | 120.20 |
| | 2 | 215.85 | | | | |
| 0.00165 | 1 | 199.25 | 199.77 | 1.20 | 0.20 | 118.69 |
| | 2 | 200.28 | | | | |
| 0.001125 | 1 | 187.34 | 187.92 | 1.12 | 0.12 | 111.05 |
| | 2 | 188.50 | | | | |
| 0.00051 | 1 | 176.25 | 176.63 | 1.06 | 0.06 | 112.39 |
| | 2 | 177.00 | | | | |
| 0.00 | 1 | 166.57 | 167.05 | - | - | - |
| | 2 | 167.53 | | | | |

- Plot curve between reduced viscosity and concentration.
- $[\eta]$ is calculated from linear equation.

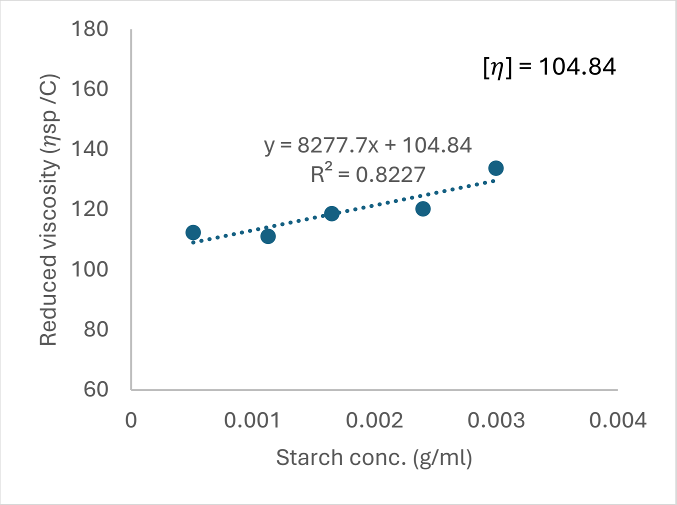


**Figure S3.** Reduced viscosity versus concentration curve.

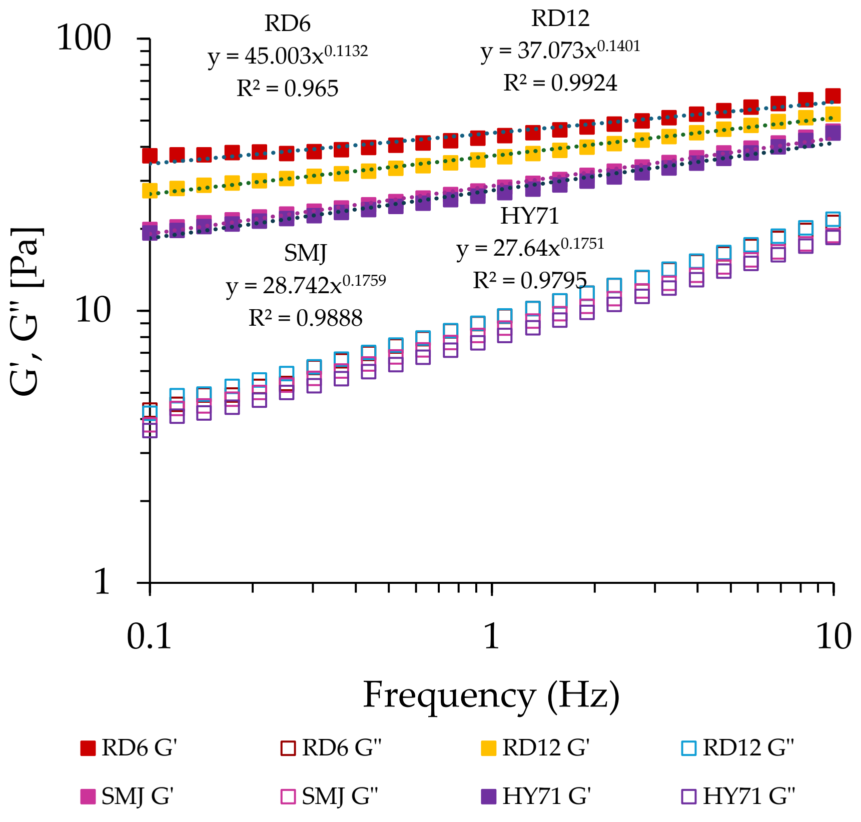


**Figure S4.** Power law index of G′ during frequency sweep of waxy rice gels.
